# An Increase in Food Insecurity Correlated with an Increase in Plasma Triglycerides among Latinx Children

**DOI:** 10.1016/j.tjnut.2023.12.024

**Published:** 2023-12-17

**Authors:** Keally Haushalter, Marissa Burgermaster, Erin Hudson, Matthew J. Landry, Shreela V Sharma, Jaimie N Davis

**Affiliations:** 1 Department of Nutritional Sciences, The University of Texas at Austin, Austin, TX, United States; 2 Department of Population Health, Dell Medical School, The University of Texas at Austin, Austin, TX, United States; 3 Department of Population Health and Disease Prevention, University of California, Irvine, Irvine, CA, United States; 4 Department of Epidemiology, Human Genetics and Environmental Sciences (UTHealth) School of Public Health, The University of Texas Health Science Center at Houston, Houston, TX, United States

**Keywords:** Food security, food insecurity, Hispanic children, Hispanic youth, cardiometabolic markers, school-based gardening intervention, longitudinal study, change in food security

## Abstract

**Background:**

Food insecurity and metabolic diseases both disproportionately affect Hispanic children. Cross-sectional studies have linked food insecurity with adverse cardiometabolic markers, including elevated plasma triglycerides and glucose concentrations. However, the association between changes in food insecurity and changes in cardiometabolic markers in children remains to be explored. Furthermore, few studies have assessed the impact of school-based nutrition interventions on household food insecurity.

**Objective:**

The objectives of this study are to assess the effect of the TX Sprouts intervention on household food insecurity and to examine the association between changes in household food insecurity and changes in cardiometabolic markers over 1 academic year.

**Methods:**

This secondary analysis used data from TX Sprouts, a cluster-randomized school-based gardening, cooking, and nutrition trial. The study enrolled 3rd–5th-grade students from 16 schools that served primarily (>50%) Hispanic families with low income in Austin, TX. Participants (*n* = 619) provided household food insecurity data and fasting lipid panels at both baseline and postintervention, ∼9 mo following.

**Results:**

There was no intervention effect on household food insecurity. Independent of the intervention, a 1-point increase in food insecurity, indicative of becoming more food insecure, was associated with a 2.61 mg/dL increase in triglycerides (*P* = 0.001; 95% CI: 1.04, 4.19) at follow-up. Children who were food insecure at baseline and became food secure at follow-up had a mean 5.05 mg/dL decrease in triglycerides compared with a 7.50 mg/dL increase in triglycerides in children who remained food insecure throughout (95% CI: −23.40, −1.71, *P* = 0.023). There were no other associations between changes in food insecurity and cardiometabolic markers.

**Conclusion:**

Although the intervention did not improve food insecurity, reductions in food insecurity over 9 mo were associated with improved cardiometabolic markers in high-risk children, emphasizing the need for interventions targeting food insecurity.

The study is registered at clinicaltrials.gov under NCT02668744 (https://classic.clinicaltrials.gov/ct2/show/NCT02668744).

## Introduction

Hispanic adults and children, compared with non-Hispanic Whites, are at a greater risk of food insecurity and are more likely to have poor cardiometabolic health [[1], [2], [3], [4]]. Among adults, cross-sectional studies have found food insecurity to be positively associated with higher rates of obesity, metabolic syndrome, plasma triglycerides, systolic blood pressure, fasting glucose, and diabetes [[5], [6], [7], [8]]. Few studies have analyzed the impact of food insecurity on children’s cardiometabolic health. Cross-sectional studies have found that children with low food security have poor glycemic control, increased insulin resistance, higher plasma triglycerides, elevated fasting glucose, and greater metabolic syndrome occurrences compared with children with high food security [[9], [10], [11]]. However, these cross-sectional studies fail to capture changes in food security status [12], and how those changes may influence changes in a child’s cardiometabolic health.

Because children’s metabolic health has been associated with their health as an adult [13], many nutrition interventions aim to improve children’s metabolic markers, yet few attempt to accomplish this by enhancing food security. The few experimental studies examining the effect of nutrition interventions on household food security yield mixed results [[14], [15], [16], [17]]. Brighter Bites, a school-based food-coop intervention that provided fresh produce and nutrition education to primarily low-income Hispanic children, decreased household food insecurity rates from 70.0% to 56.9% immediately postintervention, and further decreased to 44.4% at the 2-y follow-up [14,15]. Although Brighter Bites showed the ability of a nutrition intervention to improve food security status, it was a quasi-experimental nonrandomized study. In contrast, a year-long nutrition intervention that provided low-income children with free food increased household food insecurity [16]. The authors posit that this counterintuitive result was because providing food made the parents and children feel more food insecure because their need for food was identified and thereby influenced how they responded to post-test measures of food security [16]. Another nutrition intervention that provided children with food, nutrition education, and money to spend on fresh produce showed no effect on household food security [17]. Given the mixed outcomes nutrition interventions have on improving household food security, there is a need to assess if a nutrition randomized control trial affects food security in a high-risk population.

In this study, we assessed the effect of a recently completed school-based gardening, nutrition cluster-randomized control trial (TX Sprouts) on food insecurity in participating families. The main outcomes of TX Sprouts have been previously reported [18]. In brief, TX Sprouts increased vegetable intake, diet quality, and academic performance as well as improved circulating lipids and glycemic control [[18], [19], [20], [21]]. Although the effects of the TX Sprouts intervention on cardiometabolic outcomes have been previously reported [18], the intervention’s impact on household food insecurity as a secondary outcome remains to be seen. Furthermore, the association between changes in food insecurity and cardiometabolic outcomes has yet to be explored. We analyzed the following two secondary outcomes: *1*) the effects of the intervention on household food security status compared with the control and *2*) how changes in household food security status over 1 academic year, independent of the intervention group, were associated with changes in children’s cardiometabolic outcomes.

## Methods

### Description of study

Baseline and postintervention data from TX Sprouts, a cluster-randomized school-based gardening, cooking, and nutrition trial, was used. The methods and main outcomes of TX Sprouts have been published elsewhere [[18], [19], [20], [21], [22]]. Briefly, TX Sprouts targeted 3rd—5th grade students and their parents from 16 public elementary schools in the Austin, Texas, area that met the following criteria: *1*) a high proportion of Hispanic youth (>50%), determined by the census; *2*) located within 60 miles of the University of Texas at Austin; *3*) more than half of the students participating in the free and reduced-priced lunch (FRL) program; and *4*) no previous or existing gardening program. Seventy-three public schools met the criteria and the first 16 schools that agreed to participate were randomly assigned into 1 of 3 waves of data collection that occurred between August 2016 and October 2018. Eight schools were randomly assigned to receive the intervention and the other 8 schools were randomly assigned to receive the control, a delayed intervention the following academic year. There was no statistical difference in the proportion of Hispanic children or the proportion who participated in the FRL program in the schools that participated compared with those who did not. In all the schools, enrollment was determined by distance from residency.

### Study recruitment

All 3rd–5th grade students and their parents were recruited to participate in the study through information tables at “Back to School” and “Meet the Teachers” events, flyers that were sent home with the students, and announcements made by teachers at the beginning of the academic year.

### Ethics

To be included in the study, both parents and students signed a written informed consent and assent, respectively. The study was approved by the Institutional Review Board of The University of Texas at Austin as well as the participating school districts’ review boards.

### Data collection

Anthropometrics relevant to the analysis included height, weight, and body fat percentage of the children. Height was measured to the nearest 0.1 cm using a free-standing stadiometer that was mounted against the wall (Seca). Weight and body fat percentage were evaluated using the Tanita Body Fat Analyzer (model TBF 300). BMI (kg/m^2^), BMI percentiles, and BMI *z*-scores were determined using CDC age- and gender-specific values [23].

At both baseline and postintervention, students could choose to complete an optional fasting blood draw. Parents and students were informed that participating in the blood draw was not required and students who did not participate would still be allowed to participate in the TX Sprouts program. Parental consent and child assent were collected before the blood draw. Blood draws were collected at school by a certified phlebotomist or nurses with experience drawing blood in children with obesity. To remind students to fast for the blood draw, children and their parents received text messages and flyers that informed students to not eat or drink anything other than water the night before. Only students who completed the baseline fasting blood draw were eligible to complete the fasting blood draw postintervention. As an incentive to participate, children received $20 at both baseline and postintervention.

After the blood was collected, it was transported on ice to the University of Texas at Austin laboratory and was centrifuged, aliquoted, and stored at −80°C until all 3 waves on the study were completed. After all waves were completed, the blood was analyzed for total cholesterol, HDL cholesterol, and triglycerides using the Vitros chemistry DT slides (Ortho Clinical Diagnostics Inc.). Using the Friedewald equation, LDL cholesterol was calculated [24].

At baseline and the end of the intervention, as part of the TX Sprouts curriculum, students completed a 12-page questionnaire that included items regarding their demographics and dietary behaviors. Parents were sent a similar 12-page questionnaire with questions about their demographics as well as the 8-item child-referenced questions of the USDA Household Food Security Survey Module (HFSSM) [25]. Parents were instructed to complete the survey at home and return it to the school with their child in a sealed envelope. As an incentive to complete the questionnaire, parents who returned a completed survey received a $15 gift card to a local grocery store. The questionnaires were provided in both English and Spanish. There were no statistical sociodemographic differences between those who reported speaking Spanish only and those who spoke English.

### Assessment of household-reported food security

Household-reported food insecurity was assessed by the USDA HFSSM that the parents completed [25]. The response categories consisted of “a lot,” “sometimes,” and “never.” The responses were coded and summed as outlined by the USDA Economic Research Service [25]. Positive responses, “a lot” and “sometimes,” were encoded as 1 whereas negative responses, “never,” were encoded as 0. Scores were summed and ranged between 0 and 18, in which a higher score indicates lower food security.

To assess the change in food security, a change score was calculated. The baseline score was subtracted from the postintervention score. A positive change score indicated that one became more food insecure. A negative change score indicated that one became more food secure.

To categorize food security status, a summed score of 0–2 was labeled food secure and a summed score of 3–18 was labeled food insecure on the basis of the scoring outlined by USDA [25]. Responses were categorized both at baseline and at the end of the intervention. Responses were also placed into 1 of 4 categories on the basis of their change in food security status from baseline to the end of the intervention: those who remained secure throughout the intervention (secure to secure), those who were secure at baseline and became insecure (secure to insecure), those who were insecure at baseline and became secure (insecure to secure), and those who remained insecure (insecure to insecure).

### Statistical analysis

Descriptive statistics (mean, SD, number, and percentage) compared the demographics of those in the current analytic sample compared with those who were in the TX Sprouts clinical trial but were not included in the current analytic sample. Chi-square (χ^2^) goodness-of-fit test and independent *t-*tests evaluated whether the demographics of the 2 independent groups were statistically different.

A generalized mixed model was used to assess whether the intervention influenced household food security with schools as random clusters, controlling for children’s age, sex, ethnicity, and FRL participation. Mixed-effects linear regression models assessed the relationship between changes in household food security status and changes in cardiometabolic markers, both from baseline to postintervention. All models were adjusted for the child’s age, sex, race/ethnicity (non-Hispanic White, Hispanic, non-Hispanic Black, and Other), intervention, participation in the FRL program, BMI *z*-score, body fat percentage, the respective metabolic marker at baseline, and household food security at baseline. A general linear model analyzed whether changes in lipids differed by changes in food security groups (secure to secure, secure to insecure, insecure to secure, and insecure to insecure). All data were analyzed using SPSS version 29.0.0 (IBM Corp). To account for the multiple comparisons across the lipid panel, the results of the food insecurity and lipid regression were considered significant when *P* value was <0.0125. All other results were considered significant when *P* value was <0.05.

## Results

### Participants

**FIGURE 1:**
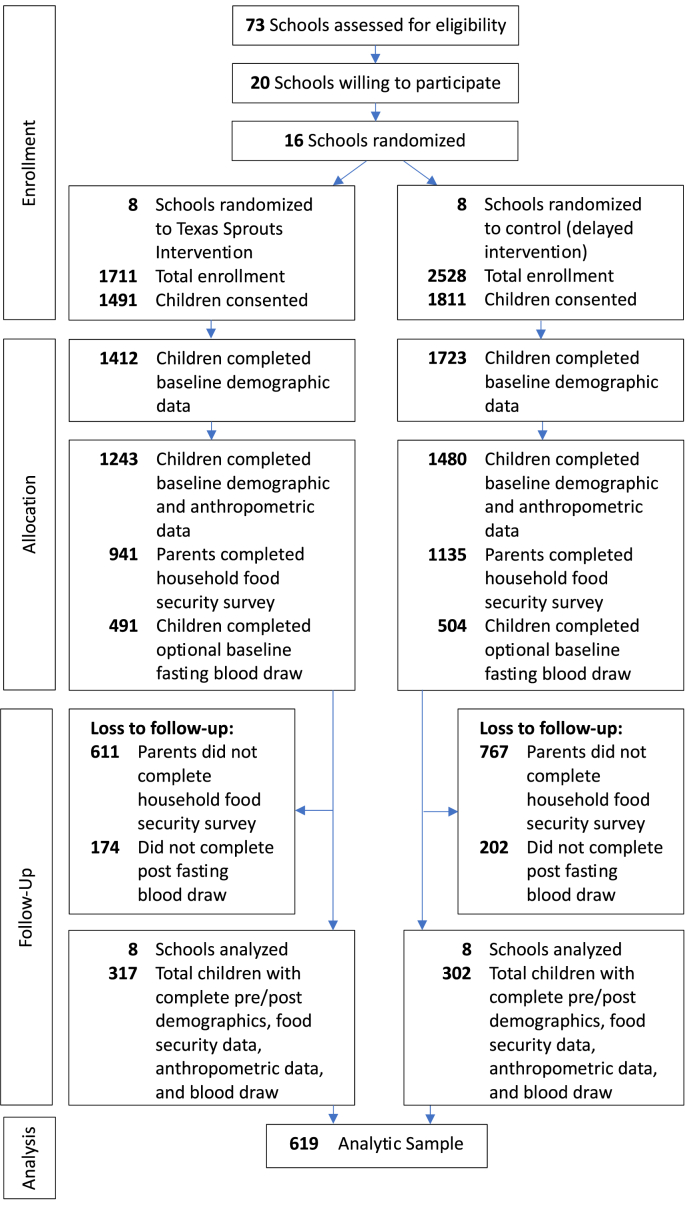
CONSORT diagram of participant flow.

Of the 4239 eligible students at the 16 elementary schools, 3302 parents consented (78%) for their child to participate in TX Sprouts. Among the 3302 children with consent, 995 (30%) completed the baseline optional fasting blood draw and 2076 parents completed the baseline (63%) household food security survey. Of those with complete baseline demographic, anthropometric, blood, and food security data, 619 children completed the same measures postintervention. The CONSORT study flow diagram is presented in Figure 1.

The baseline characteristics of participants in TX Sprouts and the analytic sample are shown in Table 1. On average, children in the analytic sample were aged 9.27 y. Children in the analytic sample had higher BMI measures and body fat percentages compared with children in the clinical trial sample (*P* < 0.001). Furthermore, compared with the clinical trial sample, children in the analytic sample were more likely to be female (*P* < 0.001), Hispanic (68.2% compared with 65%), non-Hispanic Black (10.5% compared with 8.9%), participate in the FRL program (*P* < 0.05), have higher body fat (*P <* 0.001), and have overweight/obesity (*P* = 0.001). Finally, children in the analytic sample, compared with the children in the clinical trial sample, were more food insecure (29.4% compared with 24.3%; *P* = 0.003).

**TABLE 1:** Demographic characteristics of those in the clinical trial compared with analytic sample.

Characteristics	Clinical Trial Sample (*n* = 2516)	Analytic Sample (*n* = 619)	*P* Value^1^
	<--- Mean ± SD --->		
Child age	9.23 ± 0.92	9.27 ± 0.89	0.27
Child BMI-percentile^2^	69.65 ± 29.18	73.94 ± 27.72	<0.001^7^
Child BMI *z*-score^2^	0.75 ± 1.13	0.93 ± 1.09	<0.001^7^
Child body fat percentage^2^	25.68 ± 8.85	27.02 ± 9.06	<0.001^7^
Household food security score	1.58 ± 2.54	2.07 ± 2.87	<0.001^7^

	<--- *N* (%) --->		

Child sex			<0.001^7^
Male	1204 (47.9)	281 (45.4)	
Female	1312 (52.1)	338 (54.6)	
Child ethnicity^3^			0.003^6^
Non-Hispanic White	468 (21.0)	94 (15.2)	
Hispanic	1447 (65.0)	442 (68.2)	
Non-Hispanic Black	199 (8.9)	65 (10.5)	
Other	111 (5.0)	38 (6.1)	
Child enrolled in FRL^4^			0.047^5^
Receive	1459 (68.0)	444 (71.7)	
Do not Receive	688 (32.0)	175 (28.3)	
Child BMI category^2^			0.001^6^
Normal/Underweight	1385 (55.2)	301 (48.6)	
Overweight/Obese	1122 (44.8)	318 (51.4)	
Household food security category			0.003^6^
Secure	1904 (75.7)	437 (70.6)	
Insecure	612 (24.3)	182 (29.4)	

1 Chi-square (χ^2^) goodness-of-fit test and independent *t-*test assessed the statistical difference between the clinical trial and analytic sample.

2 Clinical sample size of 2507.

3 Clinical sample size of 2225.

4 Clinical sample size of 2157.

5 Denotes *P* < 0.05

6 denotes *P* < 0.01

7 denotes *P* < 0.001.

Results of the mixed-effects regression model showed no significant changes in household food security among those in the intervention group compared with those in the control group, adjusting for baseline differences (*P* = 0.56) (data not shown). Therefore, the participants in the intervention and the control were combined into one analytic sample for all further analysis. Although the intervention did not have a significant effect, the adjusted models included intervention status as a covariate.

Table 2 shows the unadjusted and adjusted (child’s age, sex, ethnicity, intervention, FRL participation, BMI *z*-score, body fat percentage, baseline metabolic marker, and baseline food security) relationship between the continuous change in food security score and lipid biomarkers over 1 academic year. There was a significant relationship between the change in household food security and the change in child triglycerides in both the unadjusted and adjusted models (β = 2.52, *P* = 0.002, β = 2.61, *P* = 0.001, respectively). In the adjusted model, a 1-point increase, indicative of increased household food insecurity, was associated with a 2.61 mg/dL increase in child triglycerides (95% CI: 1.04, 4.19). There was no significant relationship between the change in food security and the change in HDL cholesterol, LDL cholesterol, or total cholesterol.

**TABLE 2:** Unadjusted and adjusted linear regressions of changes in metabolic markers.

	Unadjusted			Adjusted		
	*b*	95% CI	*P* value^1^	*b*	95% CI	*P* value^1^
LDL						
Intercept	15.04	[10.29, 19.79]		25.64	[11.38, 39.90]	
Age				−0.50	[−1.76, 0.77]	0.44
Sex						
Male				Ref	–	–
Female				0.42	[−2.05, 2.89]	0.74
Ethnicity						
White				Ref	–	–
Hispanic				−3.13	[−6.46, 0.21]	0.07
African American				0.85	[−3.73, 5.43]	0.72
Other				−4.11	[−9.51, 1.30]	0.14
Intervention				−0.05	[−0.32, 0.22]	0.71
Free or reduced lunch				−1.32	[−3.97, 1.33]	0.33
BMI z-score				1.53	[−0.93, 3.99]	0.22
Body fat percentage				−0.13	[−0.43, 0.17]	0.40
Baseline LDL cholesterol	−0.26	[−0.31, −0.20]	< 0.001^3^	−0.26	[−0.32, −0.21]	< 0.001^3^
Baseline food security	0.06	[−0.46, 0.58]	0.92	0.10	[−0.44, 0.63]	0.72
Change in food security	−0.54	[−1.14, 0.05]	0.07	−0.54	[−1.14, 0.06]	0.08
HDL						
Intercept	9.15	[6.67, 11.63]		12.99	[6.00, 19.97]	
Age				0.23	[−0.33, 0.78]	0.42
Sex						
Male				Ref	–	–
Female				−0.60	[−1.68, 0.49]	0.28
Ethnicity						
White				Ref	–	–
Hispanic				−1.80	[−3.26, −0.34]	0.02^4^
African American				−0.80	[−2.81, 1.21]	0.44
Other				−3.88	[−6.23, −1.52]	0.001^2^
Intervention				−0.01	[−0.12, 0.11]	0.93
Free or reduced lunch				−0.24	[−1.40, 0.92]	0.68
BMI *z*-score				−0.37	[−1.45, 0.71]	0.50
Body fat percentage				−0.06	[−0.20, 0.07]	0.34
Baseline HDL cholesterol	−0.18	[−0.22, −0.13]	<0.001^3^	−0.21	[−0.27, −0.16]	<0.001^3^
Baseline food security	−0.18	[−0.41, 0.06]	0.14	−0.16	[−0.40, 0.07]	0.17
Change in food security	−0.14	[−0.40, 0.13]	0.30	−0.16	[−0.42, 0.10]	0.23
Triglycerides						
Intercept	29.83	[22.83, 36.82]		5.84	[−29.81, 41.49]	
Age				−0.96	[−4.29, 2.37]	0.57
Sex						
Male				Ref	–	–
Female				2.05	[−4.46, 8.56]	0.54
Ethnicity						
White				Ref	–	–
Hispanic				10.46	[1.66, 19.27]	0.02^4^
African American				4.59	[−7.54, 16.71]	0.46
Other				12.64	[−1.57, 26.85]	0.08
Intervention				0.04	[−0.66, 0.75]	0.90
Free or reduced lunch				0.44	[−6.53, 7.41]	0.90
BMI *z*-score				−4.36	[−10.84, 2.13]	0.19
Body fat percentage				1.23	[0.43, 2.03]	0.003^2^
Baseline triglycerides	−0.31	[−0.38, −0.24]	< 0.001	−0.38	[−0.45, −0.30]	< 0.001^3^
Baseline food security	1.36	[−0.03, 2.76]	0.06	1.31	[−0.10, 2.71]	0.07
Change in food security	2.52	[0.94, 4.11]	0.002^2^	2.61	[1.04, 4.19]	0.001^2^
Total cholesterol						
Intercept	33.55	[25.26, 41.84]		42.32	[23.90, 60.73]	
Age				−0.56	[−2.09, 0.96]	0.47
Sex						
Male				Ref	–	-
Female				0.49	[−2.49, 3.47]	0.75
Ethnicity						
White				Ref	–	–
Hispanic				−3.02	[−7.05, 1.02]	0.14
African American				1.48	[−4.04, 7.01]	0.60
Other				−5.37	[−11.88, 1.14]	0.11
Intervention				−0.06	[−0.38, 0.27]	0.73
Free or reduced lunch				−1.40	[−4.60, 1.79]	0.39
BMI *z*-score				0.08	[−2.89, 3.05]	0.96
Body fat percentage				0.01	[−0.36, 0.37]	0.98
Baseline cholesterol	−0.26	[−0.31, −0.21]	< 0.001^3^	−0.26	[−0.31, −0.21]	< 0.001^3^
Baseline food security	0.14	[−0.49, 0.77]	0.66	0.18	[−0.47, 0.82]	0.59
Change in food security	−0.14	[−0.85, 0.58]	0.71	−0.14	[−0.86, 0.58]	0.70

1 Mixed-effects linear regression models were used to assess statistical significance. Unadjusted includes baseline food security and baseline of the respective metabolic marker. Adjusted includes child’s age, sex, ethnicity, intervention, free or reduced lunch participation, BMI *z*-score, body fat percentage, baseline metabolic marker, and baseline food security. *N* = 619.

2 Denotes *P* < 0.01.

3 Denotes *P* < 0.001.

4 Denotes *P* <0.05.

**FIGURE 2:**
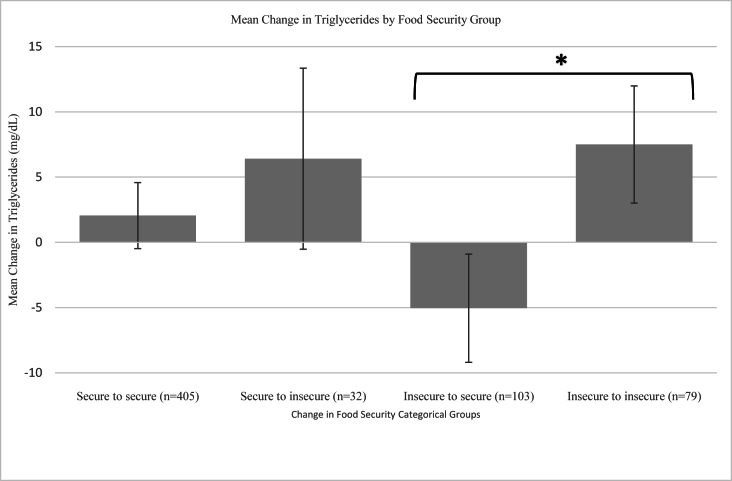
Average change in triglycerides by categorical food security groups from baseline to postintervention. Legend: ∗ denotes a significant difference in change in triglycerides between categorical food security groups (*P* = 0.02), assessed by a general linear model, controlling for covariates. Whiskers represent the standard error of the mean.

To contextualize, Figure 2 presents the average pre- and postintervention triglyceride values by the 4 categorical food security groups (secure to secure, secure to insecure, insecure to secure, and insecure to insecure). In the analytic sample, 405 (65.4%) children remained food secure, 32 (5.2%) children changed from food secure to food insecure, 103 (16.6%) children changed from food insecure to food secure, and 79 (12.8%) children remained food insecure. There was a significant difference in the change in triglycerides between children who were food insecure at baseline and became food secure at 9-mo follow-up compared with children who remained food insecure (β = −12.55, 95% CI: −23.40, −1.71, *P* = 0.023). Children who were food insecure at baseline but became food secure at 9-mo follow-up had a mean −5.05 mg/dL decrease in triglycerides whereas children who remained food insecure at both time points had a mean 7.50 mg/dL increase in triglycerides.

## Discussion

In this study, we analyzed the association between changes in food security with changes in plasma lipids from baseline to postintervention in primarily Hispanic low-income 3rd–5th grade children. Although the TX Sprouts intervention did not impact household food security, an increase in food insecurity, independent of the intervention, was significantly associated with an increase in children’s plasma triglycerides over 1 school year. Furthermore, there was a significant 12.55 mg/dL difference in the change of triglycerides between children in households who were food secure at baseline and became food insecure at postintervention follow-up compared with those who remained food insecure across both time points. To the best of our knowledge, this is the first study to assess how a change in food security relates to changes in lipids in primarily low-income Hispanic children.

Among TX Sprouts participants, body fat percentage was associated with elevated triglycerides. Studies have consistently found that triglycerides, but no other blood lipid, are significantly elevated for children with obesity and overweight when compared with children with normal weight [26,27]. The association between excess adiposity and elevated triglycerides is because of the inefficiency of adipose tissues to use fatty acids when expanded [28]. When an individual gains weight, their adipose tissues expand and eventually reach a point where they cannot store any more lipids [29]. In these states, fatty acids, the product of excessive caloric intake, are not efficiently sequestered by adipose tissues, and the leftover fatty acids are sent to the liver to serve as substrates for triglycerides [28]. With more substrate, hepatic triglyceride production increases, and triglycerides are excreted into the plasma, leading to an increase in plasma triglycerides [28]. However, given that baseline body fat percentage was controlled for in the model, increases in food insecurity were associated with increased triglycerides independent of body fat percentage, further emphasizing the importance food security status can have on metabolic health and the need for it to be explored further.

Poor diets have also been associated with elevated triglycerides. In a racially diverse study of 320 children (7–12 y of age), added sugar consumption was positively associated with triglycerides, but was not associated with other blood lipids [30]. In addition, a study conducted on 202 multiethnic children (7–12 y of age) found that high carbohydrate intake was positively associated with plasma triglycerides and glucose concentrations, but not with any other blood lipids [31]. One potential mechanism that could explain how dietary intake impacts triglycerides, but not other blood lipids is through an increase in hepatic de novo lipogenesis [32,33]. Hepatic de novo lipogenesis is a process in which excess carbohydrates are converted into fatty acids in the liver [34] The liver then incorporates these fatty acids into triglycerides, which are excreted into the plasma [34]. Excess carbohydrate intake, specifically added sugar intake, can increase hepatic de novo lipogenesis, which subsequently increases plasma triglycerides [35]. Given this mechanism, plasma triglycerides may be an early indicator of an adverse diet, whereas other plasma lipids may not become elevated until an adverse diet is prolonged.

Elevated plasma triglycerides, in the absence of other abnormal lipids, have negative health implications. A longitudinal study found that having elevated triglycerides as a child was associated with increased carotid intima-media thickness as an adult [36], which has been associated with coronary artery disease [37], and cardiovascular events [38]. Furthermore, elevated plasma triglycerides have been associated with type 2 diabetes, metabolic syndrome, obesity, and chronic kidney disease [39]. Given the association between food insecurity and plasma triglycerides as seen in this study, improving children’s food security could be important to help prevent future adverse health outcomes.

Suboptimal diets often occur with food insecurity. In the TX Sprouts study, lower food security was associated with lower diet quality, as measured by the Healthy Eating Index-2015 at baseline [40]. Specifically, food-insecure children had lower seafood and plant protein, greens and beans, and added sugar component scores compared with food secure children [40]. The analysis to examine the relationship between changes in food security and diet quality is currently underway. Furthermore, a study of 3605 4th- and 5th-grade children in high-poverty schools found that those who were food insecure consumed more total energy, fat, sugar, and fiber and fewer vegetables than those who were food secure [41]. In addition, studies have found that compared with children in food-secure households, children in food-insecure households consumed significantly fewer fruits, vegetables, dairy, and protein foods, and significantly more refined grains [42,43]. Considering that children’s food consumption is mainly driven by their parents [44], it is important to understand what drives adults’ food choices. A common determinant for food choice among food-insecure adults is the cost of food and the time it takes to prepare a healthy meal [[45], [46], [47], [48]]. Low-cost and readily available foods are often high in carbohydrates, fat, and energy [49]. The overconsumption of carbohydrates and calories could lead to an increase in plasma triglycerides through the mechanisms previously mentioned.

There are multiple potential reasons why TX Sprouts did not improve household food security among participants. Studies have shown that incorporating parents in the intervention and improving access to and availability of healthy food is effective in improving dietary intake [[50], [51], [52], [53], [54]] and reducing food insecurity [55]. However, whereas TX Sprouts included monthly parent nutrition and gardening education lessons, there was low parental engagement (7.1% of participating parents attended 1 of the 9 offered parent lessons) [18]. Therefore, the lack of parental engagement might be why the intervention did not reduce household food insecurity. Furthermore, TX Sprouts did not provide food for children to bring home. Many of the studies successful in reducing food security provided children with food for their families [14,15]; however, this was not a part of the TX Sprouts study design.

Several limitations need to be mentioned. There were significant differences between those who were in the analytic sample compared with those in the clinical trial. The individuals in the analytic sample were more likely to be a low-income, minority, food insecure, and overweight or obese. One explanation for this is that parents of children with overweight/obesity may have been more concerned about their child’s health and opted to receive the free diabetes screening, as this was a benefit of participating in the blood draw. In addition, individuals of Hispanic origin or who have a low income may have less access to healthcare, which could have encouraged them to sign up for the free diabetes screening test. Regardless, the subsample with blood draws represented a high-risk group; thus, the results cannot be generalized to healthier pediatric populations of higher socioeconomic status. In addition, there was a substantial proportion (∼38%) of students who completed the baseline blood draw but did not complete the follow-up. Although the reasons for drop-out were not officially collected, several children and parents reported that they did not think they needed another diabetes screening at follow-up (9 mo after the baseline), which was their main motivation to complete the baseline blood draw. In addition, because the blood draw took place before the school day began, parents had to drive their children to school early for them to participate in the blood draw. This served to be a challenge for parents as most children typically rode the bus to school so driving their child to school was not a part of their typical morning routine. Furthermore, the sample sizes of the 4 categorical food security groups differed, with those who remained food secure being relatively large (*n* = 405) and those who became food insecure quite small (*n* = 32). However, despite the differing sample sizes, there was still a significant difference observed between those who remained food insecure and those who were food secure and became food insecure.

Although the current TX Sprouts intervention resulted in numerous health benefits and academic performance [[18], [19], [20], [21]], it did not reduce household food insecurity as hypothesized. Interventions that focus on improving accessibility and availability of healthy, fresh foods to children and their parents may have significant effects on reducing household food insecurity [56,57]. However, this study does highlight how changes in food insecurity over 1 school year can alter cardiometabolic markers, which emphasizes the need for more interventions targeting food insecurity.

## Authors contributions

The authors’ responsibilities were as follows – KH: Analyzed data, performed the statistical analysis, wrote, and edited the original manuscript; KH, MB, JDN: formulated the research question, supervised, and assisted data analysis; all authors: interpreted the data, edited, read, and approved the final manuscript.

## Conflict of interest

The authors report no conflicts of interest.

## Funding

Funding was provided by the National Heart, Lung, and Blood Institute (NHLBI) (grant number R01HL123865).

## Data availability

Data described in this article, code book, and analytic code will be made available upon request.
